# Epidemiology and likelihood of asymptomatic malaria among community dwellers in the Fanteakwa south district of Ghana

**DOI:** 10.1016/j.parepi.2024.e00378

**Published:** 2024-09-05

**Authors:** Enoch Aninagyei, David Adedia, Gifty Larbi, Stella Omane Acheampong, Margaret Nyarko, George Abeiku Abbew, Isaac Tuwarlba, Desmond Omane Acheampong

**Affiliations:** a School of Basic and Biomedical Sciences, Department of Biomedical Sciences, University of Health and Allied Sciences, PMB 31 Ho, Volta Region, Ghana; b School of Basic and Biomedical Sciences, Department of Basic, University of Health and Allied Sciences, PMB 31 Ho, Volta Region, Ghana; c School of Allied Health Sciences, Department of Biomedical Sciences, University of Cape Coast, Central Region, Ghana; d School of Physical Sciences, Department of Statistics, University of Cape Coast, Central Region, Ghana; e Ghana Health Service, Fanteakwa South District Health Directorate, Eastern Region, Ghana

**Keywords:** Asymptomatic malaria, Epidemiology, Mass strategy, Mass testing, Mass drug administration, Mass relapse prevention, Fanteakwa south district

## Abstract

**Background:**

Data on the asymptomatic burden of malaria in endemic areas is essential for Ghana's malaria elimination efforts. Consequently, the situation of asymptomatic malaria in the Fanteakwa South District (FSD) is determined in this study. The FSD is predominantly forested with more rural than peri-urban communities. Additionally, artisanal mining is prevalent in the district. Despite that the forgoing could promote high incidence of malaria, the burden of asymptomatic malaria and associated factors in the district have never been determined.

**Methods:**

This community-based cross-sectional study was conducted in four randomly selected communities in the FSD in the Eastern region of Ghana. The participating households were systematically selected, of which one household member was randomly enrolled in the study. With prior consent, 2 mL of whole blood was collected from the participants. Subsequently, the study variables were obtained from the enrolees using a structured questionnaire. The malaria status of the enrolled participants was determined using the CareStart™ malaria rapid diagnostic test kit (mRDT) (USA). The multiple logistic regression model was used to fit the model to predict the groups at risk of *P. falciparum* infection in the district.

**Results:**

In total, 412 study participants were enrolled. The overall prevalence of asymptomatic malaria in the district was 43.4 % (179/412). The prevalence rate was 36.9 %, 27.7 %, 50 % and 58.8 % (<0.001) respectively for the Dwenase, Bosusu, Nsutam and Osino communities. Living at Bosusu (*p* = 0.045, AOR = 0.23, 95 % CI: 0.05–0.96), Dwenase (*p* < 0.001, AOR = 0.12, 95 % CI: 0.04–0.30) and Nsutam (p < 0.001, AOR = 0.19, 95 % CI: 0.08–0.45) were less likely to contract malaria compared to Osino dwellers. Furthermore, pregnant women (*p* = 0.024, COR = 0.35, 95 % CI: 0.14–0.9) and individuals who do not share mosquito nets with others (*p* = 0.017, COR = 0.47, 95 % CI: 0.25–0.88) were less likely to contract malaria. Moreover, being an adolescent (*p* = 0.048, COR = 1.93, 95 % CI: 1.00–3.73), living in mining communities (*p* = 0.002, COR = 1.97, 95 % CI: 1.27–3.05), being nocturnally active (*p* = 0.001, AOR = 4.64, 95 % CI: 1.97–11.31), living in a medium quality house (*p* = 0.031, AOR = 2.31, 95 % CI: 1.09–5.00), schooling in the district (*p* < 0.001) and body temperature above >37.5 °C (<0.001), were predictors of asymptomatic malaria.

**Conclusions:**

The burden of asymptomatic malaria is high in the Fanteakwa South district. In this context, the implementation of the ‘mass strategy’ recommended by the World Health Organization will play a key role in eliminating malaria in the district.

## 1 Background

Ghana has declared its intention to eliminate malaria among its citizens by 2028, by which time malaria incidence and mortality would have reduced by 50 % and 90 % respectively *(*Aidoo et al., 2024). One of the three-prong approach recommended by the World Health Organization (WHO) to achieve malaria elimination is the ‘mass strategy’. This strategy involves mass testing, mass drug administration, and mass relapse prevention (WHO, 2024). Active disease surveillance is a core activity in mass testing, which is aimed at detecting asymptomatic or subclinical conditions for management. This effort is a cardinal step in the effort to eradicate the disease. Malaria surveillance is an important epidemiological tool that is used to describe the current burden and epidemiology of the disease. Additionally, malaria surveillance aids to monitor disease trends and, finally, to identify outbreaks and emerging parasites (Murray and Cohen, 2017).

Ghana is holoendemic for malaria (Nkrumah et al., 2011), however, there are high and low focal transmission zones in the country. However, malaria endemicity in all zones in Ghana is not known. To eliminate malaria in an endemic region, it is imperative that the WHO ‘mass strategy’ is implemented in the medium and high burden zones. It is for this reason that this study seeks to determine the situation of asymptomatic malaria in the Fanteakwa south district.

The Fanteakwa South district is one of the 26 districts in the Eastern region of Ghana. The district is predominantly forested and more than 75 % of the communities are rural. A recent report indicated that more than half of the communities in the districts have been overtaken by artisanal miners (FSDA, 2021). The common sight of degrading forests, farmlands and the pockets of stagnant water in many communities in the district could encourage breeding mosquitoes. In a previous study carried out in some mining communities in Ghana, it was found that both the S and M forms of *Anopheles gambiae* survived in polluted water bodies in mining communities (Hunt et al., 2011). In mining areas, about 9 % of these vectors were infected with *Plasmodium falciparum.* The study also detected West African *kdr* mutation in very high frequencies in mosquitoes. This mutation makes mosquitoes resistant to pyrethroids, carbamates, organophosphates, and organochlorines insecticides (Hunt et al., 2011).

Despite these observations in the Fanteakwa South district, the prevalence and factors likely to be associated with asymptomatic malaria in the district are not known. Malaria is routinely diagnosed in Ghana using microscopy and rapid diagnostic test (RDT) kits. However, microscopy has little (Aninagyei et al., 2019) or no (Bousema et al., 2014) value regarding asymptomatic malaria. This is because most of the *P. falciparum* infection cases in these communities are characterized by low parasitaemia (Abukari et al., 2019). Compared to microscopy, the malaria RDT kit is relatively sensitive, user-friendly, cheap, and suitable for various diagnostic settings (Aninagyei, 2020). In a reactive malaria case detection study in Ghana, the RDT was found to be 76.5 % sensitive in detecting asymptomatic malaria *(*Aidoo et al., 2024). Therefore, RDT was used to carry out this epidemiology study to determine baseline data for malaria elimination efforts in the district.

## 2 Methods

### 2.1 Study design, study sites, and study population

This community-based cross-sectional study was conducted in the Fanteakwa South district (FSD) of the Eastern region of Ghana. Four communities were randomly selected using stratified random sampling. To do this, the district was divided into four quadrants. From each quadrant, a community was randomly selected for this study. Based on this sampling technique, the Bosusu, Dwenase, Nsutam, and Osino communities were selected for the study. Community members who were permanent residents of the district participated in the study. Additionally, community dwellers that had fever, chills and/or headache and had tested positive for malaria in the past month and those with active malaria were excluded from the study. Participants were recruited into the study from 12 July to 5 October 2023.

### 2.2 Sample size calculation

A sample size of 384 was determined to be selected for the study. The sample size was calculated using the Cochrane's formula which is *N = z*^*2*^ *p(1-p)/e*^*2*^ where N is the sample size, *z* is the standard value of 1.96 corresponding to confidence level at 95 % and *e* is the error margin at 5 %. Because the prevalence of malaria in the Fanteakwa South district is unknown, the prevalence (p) was estimated at 50 %. The sample size was increased by 5 % to accommodate incomplete and invalid data. Therefore, 403 individuals were expected to participate in this study, thus 100 from each community.

### 2.3 Community entry procedures

The researchers engaged community members after rigorous community entry procedures. First, the researchers contacted the Fanteakwa South District Health Administration (FSDHA) for the details of the community-based surveillance volunteers (CBSV) located in each community. CBSV introduced the team to the local government representatives (popularly known as the assembly members) located in each electoral area of the study community. Finally, community leaders were informed about the study before the heads of households were engaged.

### 2.4 Households and participant selection

From each study site, 100 households were selected. Households were systematically selected. This was done by dividing the number of households in each study community by 100 to obtain an n^th^. Using the house of the assembly member as the starting point, the n^th^ household was selected, until about 100 households were selected. The number of households for each of the study sites was obtained from the Fanteakwa South District Assembly (FSDA). Consent to participate in the study was obtained from the head of the household. Subsequently, the number of households was counted as N. The N-1 papers were marked ‘NO’ and only one marked ‘YES’. The papers were placed in an opaque box. Members of the household were asked to select one paper, in turn. For children less than four years of age and those with poor vision, available adults living in the same household selected for them. The only person that picked ‘YES’ in each household were selected for the study. Written consent was obtained from such people while, for children under 16 years of age, parental consent was sought on their behalf, before blood samples were collected and subsequently interviewed. Guardians were interviewed on behalf of children under 16 years of age.

### 2.5 Phlebotomy

A phlebotomist, certified by the Ghana Health Service, obtained 2 mL of whole blood from each participant. The phlebotomist was hired from the health care facility in each community. For participants with visible antecubital fossa veins, venepuncture was performed after double disinfection of the skin with 70 % alcohol followed by a povidone‑iodine solution. For those without palpable veins, fingertip sample collection was carried out using a BD Microtainer contact-activated lancet (Dublin, Ireland). Blood was placed in a tube containing CPDA-1 anticoagulant in a ratio of 1 part of the anticoagulant to 7 parts of blood (Almokhtar and Ali, 2021). Blood samples were stored at 4–8 ^°C^ in a nearby health center prior to collection for analysis.

### 2.6 Study variables

A structured questionnaire was used to obtain relevant study variables. The dependent variable was the result of the malaria rapid diagnostic test, whereas the independent variable was the demographic characteristics of the enrolees. Other independent variables were the presence of mining activity, type of mining activity, mosquito net ownership, source of mosquito net, mosquito net usage, number of persons per net, previous night mosquito net usage, farm visitation during the previous week and housing quality. The quality of the housing was assessed using a composite assessment score designed specifically for this study. Six variables were used to assess the quality of the building. They were type of building material (mud / clay = 0, cement = 1), type of roof (grass lined = 0, metal roofing sheets = 1), type of floor (clay = 0, Concrete = 1, tiles = 2), ceiling (no = 0, yes = 1), windows with nets (no = 0, yes = 1) and availability and regular use of the electric ceiling fan (no = 0, yes = 1). The composite scores for low, medium and desirable housing quality were 0–2, 3–4 and 5–6, respectively.

### 2.7 Diagnosis of malaria

The diagnosis of asymptomatic malaria was performed by detecting *P. falciparum* specific histidine-rich protein II (PfhrpII) in whole blood. This was done using the CareStart™ malaria rapid diagnostic test (mRDT) kit (USA). Using this kit, 5 μL of whole was used. The test result was at 20 min after adding the last drop of the buffer.

### 2.8 Assessing the diagnostic sensitivity of CareStart™ rapid diagnostic test kit

To assess the confidence of the CareStart™ mRDT kit to diagnose asymptomatic malaria, the limit of detection (LOD) was assessed. This was done by selecting three blood samples with known parasitemia (32,808 parasites/ μL, 18,990 parasites/ μL and 9007 parasites/ μL) from patients with clinical malaria. Blood samples were serially diluted using phosphate buffered saline and subsequently tested using mRDT. The mean LOD for CareStart™ mRDT was 94 parasites/μL.

### 2.9 Statistical analysis

The SPSS and R statistical package were used for the statistical analysis. Chi-square and Fishers exact tests were used to assess associations between possible predictors and *P. falciparum* infection in bivariate analyses. Mann Whitney test was used to compare temperature of those who have and those who do not have malaria. Multiple logistic regression model was used to fit the model for predicting *P. falciparum* infection while controlling for other factors. The model was assessed using model fit measures including likelihood ratio test, Nagelkerke pseudo-R-squared, and receiver characteristic analysis of the model. A test with a *p* ≤ 5 % was considered significant.

### 2.10 Ethics approval and obtaining informed consent

Ethics approval for the study was obtained from the Ghana Health Service Ethics Review Committee. The approval number was GHS-ERC: 007/02/22. Written consent was obtained from each study participant.

## 3 Results

### 3.1 Descriptive characteristics of study participants

In total, 412 study participants selected from four communities participated in the study. Out of the total, 101 (24.5 %), 102 (24.8 %), 103 (25 %), and 106 (25.7 %) enrolled were selected from Bosusu, Osino, Dwenase, and Nsutam, respectively. The majority of the participants were females (60.2 %), belonging to the Akan ethnic group (69.9 %), and belonging to the Christian religion (89.8 %). Furthermore, the number of adult participants (18–65 years) (49 %) was more than the total number of children, adolescents, and older adults, separately. However, the number of minors (42 %) was greater than the total number of married, single, and previously married participants. Furthermore, the number of participants who have stayed continuously in the community between 1 and 9 years (40 %) was higher than those who had stayed in the community for more than 9 years (Table 1).

**Table 1:** Prevalence and demographic factors associated with malaria among study participants.

	*P. falciparum* status				
	Positive (%)	Negative (%)	Total (%)	*p*-value	COR (95 % CI)
Overall	179 (43.4 %)	233 (56.6)	412 (100)		
*Study community*				<0.001	
Bosusu	28 (27.7 %)	73 (72.3 %)	101 (24.5)	<0.001	0.27 (0.15–0.48)
Dwenase	38 (36.9 %)	65 (63.1 %)	103 (25)	0.002	0.41 (0.23–0.72)
Nsutam	53 (50.0 %)	53 (50.0 %)	106 (25.7)	0.202	0.7 (0.41–1.21)
Osino	60 (58.8 %)	42 (41.2 %)	102 (24.8)		1

*Gender*					
Female	102 (41.1 %)	146 (58.9 %)	248 (60.2)	0.243	0.79 (0.53–1.18)
Male	77 (47.0 %)	87 (53.0 %)	164 (39.8)		1

*Pregnancy status*					
Positive	6 (21.4 %)	22 (78.6 %)	28 (11.3)	0.024	0.35 (0.14,0.9)
Negative	96 (43.6 %)	124 (56.7 %)	220 (88.7)		1

*Ethnic group*				0.696	
Ewe	18 (54.5 %)	15 (45.5 %)	33 (8)	0.169	1.66 (0.8–3.42)
Ga-Adangbe	4 (36.3 %)	7 (63.7 %)	11 (2.7)	0.767	0.79 (0.23–2.75)
Krobo	21 (45.6 %)	25 (54.4 %)	46 (11.2)	0.643	1.16 (0.62–2.17)
Northern descent	15 (44.1 %)	19 (55.9 %)	34 (8.3)	0.814	1.09 (0.53–2.23)
Akan	121 (42.0 %)	167 (58.0 %)	288 (69.9)		1

*Religion*				0.72	
Christian	161 (43.5 %)	209 (56.5 %)	370 (89.8)	0.497	1.93 (0.37–10.05)
Moslem	16 (45.7 %)	19 (54.3 %)	35 (8.5)	0.679	2.11 (0.36–12.35)
Traditional	2 (28.6 %)	5 (71.4 %)	7 (1.7)		1

*Marital status*				0.017	
Single	47 (43.1 %)	62 (56.9 %)	109 (26.5)	0.118	1.54 (0.9–2.63)
Minor (< 18 years)	89 (51.4 %)	84 (48.6 %)	173 (42)	0.002	2.15 (1.32–3.49)
Widow/widower	4 (33.3 %)	8 (66.7 %)	12 (2.9)	1	1.01 (0.29–3.57)
Married	39 (33.0 %)	79 (67.0 %)	118 (28.6)		1

*Developmental stage*^1^				<0.001	
Adolescent	34 (64.2 %)	19 (35.8 %)	53 (12.9)	0.048	1.93 (1.00–3.73)
Adult	77 (38.1 %)	125 61.9 %)	202 (49)	0.072	0.67 (0.43–1.04)
Older adult	5 (19.2 %)	21 (80.8 %)	26 (6.3)	0.007	0.26 (0.09–0.72)
Children	63 (48.1 %)	68 (51.9 %)	131 (31.8)		1

*Duration of stay (years)*				0.024	
10–19	67 (55.4 %)	54 (44.6 %)	121 (29.4)	0.010	1.86 (1.16–2.99)
20–29	16 (33.3 %)	32 (66.7 %)	48 (11.7)	0.403	0.75 (0.38–1.48)
30–39	8 (32.0 %)	17 (68.0 %)	25 (6.1)	0.445	0.71 (0.29–1.73)
40+	22 (41.5 %)	31 (58.5 %)	53 (12.9)	0.846	1.07 (0.57–2.00)
1–9	66 (40.0 %)	99 (60.0 %)	165 (40)		1

1 Minor is a child less than marital age, according the laws of Ghana; ^2^ The classification of developmental stages was done according to the age designations of the American Medical Associations. Children (1 to 12 years), adolescents (13 to 17 years), adults (18 to 65 years) and Older adult (> 65 years) (NIH, 2022). COR – crude odds ratio.

### 3.2 Prevalence of asymptomatic malaria among study variables

The overall prevalence of asymptomatic malaria in the district was 43.4 % (179/412). The prevalence rate for asymptomatic malaria was 36.9 %, 27.7 %, 50 %, and 58.8 % (*p* < 0.001) respectively for the communities of Dwenase, Bosusu, Nsutam, and Osino. The prevalence rate was relatively higher among males (47 %) compared to females (41.1 %) (*p* = 0.243). However, among the females, prevalence was higher among non-pregnant women (43.6 %) compared to pregnant women (21.4 %) (*p* = 0.024). Furthermore, the rate of asymptomatic infection was higher among adolescents (64.2 %) while among older adults (19.2 %), the rate was lower (*p* < 0.001). Community members who had stayed in the community for 10–19 years reported a higher prevalence (55.4 %) compared to those who had stayed in the communities for other durations (*p* = 0.024) (Table 1).

### 3.3 Demographic factors associated with the likelihood of asymptomatic malaria

Study community, pregnancy status, marital status, developmental stage, and duration of stay in the community associated with asymptomatic malaria. Study participants who stayed at Bosusu (p < 0.001, COR = 0.27, 95 % CI: 0.15–0.48) and Dwenase (*p* = 0.002, COR = 0.41, 95 % CI: 0.23–0.72) were less likely to contract malaria compared to those who lived in Osino. Although gender was not associated with malaria status, pregnant women were less likely to contract malaria than non-pregnant women (p = 0.024, COR = 0.35, 95 % CI 0.14–0.9). Furthermore, those who had stayed in their respective community for 10 to 19 years were more likely to contract malaria compared to those whose stay duration was less than 10 years (*p* = 0.010, COR = 1.86, 95 % CI: 1.16–2.99). Compared to children aged 1 to 12 years, adolescents (13–17 years) were more likely to contract malaria (*p* = 0.048, COR = 1.93, 95 % CI: 1.00–3.73), while older adults (> 65 years) (*p* = 0.007, COR = 0.26, 95 % CI: 0.09–0.72), were less likely to contract malaria (Table 1).

### 3.4 Likelihood of asymptomatic malaria with respect to educational levels and occupation category

Table 2 details the association and likelihood analyses of asymptomatic malaria in relation to educational levels and occupations. Compared to those who are still in school, those below formal education age (< 4 years) (*p* = 0.005, COR = 0.4, 95 % CI: 0.21–0.77), completed school (*p* < 0.001, COR = 0.31, 95 % CI: 0.2–0.49) and those without formal education (*p* = 0.011, COR = 0.3, 95 % CI: 0.12–0.79) were less likely to contract malaria. Among those with high school education, those who completed were less likely to contract malaria than those who were still in school (p < 0.001, COR = 0.23, 95 % CI 0.10–0.54). Similarly, among participants with junior high school education, those who completed were less likely to contract malaria compared to those who were still in school (p = 0.011, COR = 0.32, 95 % CI: 0.13–0.79). Additionally, compared to those who were not in the working category (students), those in farming-related jobs (*p* = 0.035, COR = 0.43, 95 % CI 0.19–0.96) and non-farming-related jobs (p < 0.001, COR = 0.46, 95 % CI 0.29–0.73) were less likely to have malaria.

**Table 2:** Association of educational and employment details of study participants with asymptomatic malaria.

	*P. falciparum* infection				
	Positive (%)	Negative (%)	Total (%)	p-value	COR (95 % CI)
*Educational levels*				<0.001	
Below formal education (<4 yrs)	19 (38.0 %)	31 (62.0 %)	50 (12.1)	0.005	0.4 (0.21–0.77)
Completed school	61 (32.4 %)	127 (67.6 %)	188 (45.6)	<0.001	0.31 (0.2–0.49)
No formal education	7 (31.8 %)	15 (68.2 %)	22 (5.3)	0.011	0.3 (0.12–0.79)
Still in school	92 (60.5 %)	60 (39.5 %)	152 (36.9)		1

*Tertiary education*					
Completed	11 (28.2 %)	28 (71.8 %)	39 (79.6)	0.702	0.59 (0.14–2.5)
Ongoing	4 (40.0 %)	6 (60.0 %)	10 (20.4)		1
*Senior high school*					
Completed	16 (28.1 %)	41 (71.9 %)	57 (57)	<0.001	0.23 (0.10–0.54)
Ongoing	27 (62.7 %)	16 (37.3 %)	43 (43)		1

*Junior high school*					
Completed	30 (38.0 %)	49 (62.0 %)	79 (73.1)	0.011	0.32 (0.13–0.79)
Ongoing	19 (65.5 %)	10 (34.5 %)	29 (26.9)		1

*Primary school*					
Completed	4 (30.7 %)	9 (69.3 %)	13 (15.7)	0.052	0.3 (0.08–1.06)
Ongoing	42 (60.0 %)	28 (40.0 %)	70 (84.3)		1

*Occupation*				0.005	
Farming related job	10 (32.3 %)	21 (67.7 %)	31 (7.5)	0.035	0.43 (0.19–0.96)
Non-farming related job	48 (34.0 %)	93 (66.0 %)	141 (34.2)	<0.001	0.46 (0.29–0.73)
Unemployed	26 (43.3 %)	34 (56.7 %)	60 (14.6)	0.205	0.68 (0.38–1.23)
School children	95 (52.8 %)	85 (47.2 %)	180 (43.7)		1

COR – crude odds ratio.

### 3.5 Malaria risk factors identified in the study communities

**Fig. 1:**
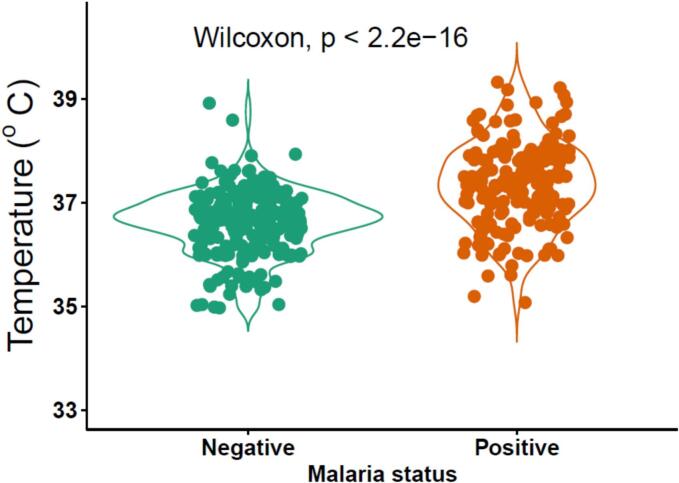
Comparison of temperature readings among those with and without malaria.

Bivariate analysis revealed that participants who lived in communities with mining activity were twice as likely to have malaria compared to those that lived in communities without mining activity (p = 0.002). Furthermore, people who used mosquito nets alone (p = 0.017, COR = 0.47, 95 % CI 0.25–0.88) were less likely to contract malaria compared to those who shared their nets with others. Furthermore, those who regularly or sometimes stayed outdoors at night were more than three times (*p* < 0.001) or more than twice (<0.001), respectively were more likely to have malaria compared to those who regularly stayed indoors at night. Those who had a medium quality of housing (*p* = 0.006, COR = 2.13, 95 % CI 1.24–3.66) were more likely to have malaria than those who had a desirable quality of housing (Table 3). Body temperature above 37.5 ^o^C was associated with the positive *P. falciparum* outcome (*p*-value<0.001) (Fig. 1).

**Table 3:** Risk factors associated with malaria transmission among community dwellers.

	*P. falciparum* infection				
	Positive (%)	Negative (%)	Total (%)	p-value	COR (95 % CI)
*Presence of mining activity*					
Yes	138 (48.4 %)	147 (51.6 %)	285 (69.2)	0.002	1.97 (1.27–3.05)
No	41 (32.3 %)	86 (67.7 %)	127 (30.8)		1

*Type of mining activity*					
Galamsey	132 (48.4 %)	141 (51.6 %)	273(95.8)	0.911	0.94 (0.30–2.98)
Small scale	6 (50.0 %)	6 (50.0 %)	12 (4.2)		1

*Mosquito net ownership*					
Yes	78 (41.7 %)	109 (58.3 %)	187 (45.4)	0.517	0.88 (0.59–1.30)
No	101 (44.9 %)	124 (55.1 %)	225 (54.6)		1

*Source of mosquito net*				1.000	
Bought from a chemical shop	2 (33.3 %)	4 (66.7 %)	6 (3.2)	1.000	0.69 (0.12–3.88)
Bought from the open market	1 (50.0 %)	1 (50.0 %)	2 (1.1)	1.000	0.69 (0.06–7.79)
Health service provider	75 (41.9 %)	104 (58.1 %)	179 (95.7)		1

*Mosquito net usage*					
I use it alone	21 (30.4 %)	48 (69.6 %)	69 (36.9)	0.017	0.47 (0.25–0.88)
Shared	57 (48.3 %)	61 (51.7 %)	118 (63.1)		1

*Number of persons per one net*				0.916	
Two persons	19 (46.3 %)	22 (53.7 %)	41 (34.7)	0.713	0.86 (0.40–1.89)
More than two persons	5 (45.4 %)	6 (54.6 %)	11 (9.3)	0.780	0.83 (0.23–3.00)
One person	33 (50.0 %)	33 (50.0 %)	66 (55.9)		1

*Previous night ITN usage*					
Yes	57 (40.7 %)	83 (59.3 %)	140 (74.9)	0.633	0.85 (0.44–1.66)
No	21 (44.7 %)	26 (55.3 %)	47 (25.1)		1

*Prolonged nocturnal outdoor activity*				<0.001	
Yes	55 (53.4 %)	48 (46.6 %)	103 (25)	<0.001	3.15 (1.80–5.52)
At times	92 (48.7 %)	97 (51.3 %)	189 (45.9)	<0.001	2.61 (1.59–4.28)
No	32 (26.7 %)	88 (73.3 %)	120 (29.1)		1

Farm visitation during the previous week					
Yes	53 (47.7 %)	58 (52.3 %)	111 (26.9)	0.285	1.27 (0.82–1.97)
	126 (41.9 %)	175 (58.1 %)	301 (73.1)		1

Housing quality				0.011	
Medium	40 (59.7 %)	27 (40.3 %)	67 (16.3)	0.006	2.13 (1.24–3.66)
Low	24 (36.9 %)	41 (63.1 %)	65 (15.8)	0.539	0.84 (0.48–1.47)
Desirable	115 (41.1 %)	165 (58.9 %)	280 (68)		1

COR – crude odds ratio.

### 3.6 Predictors of asymptomatic malaria in the Fanteakwa South District

**Fig. 2:**
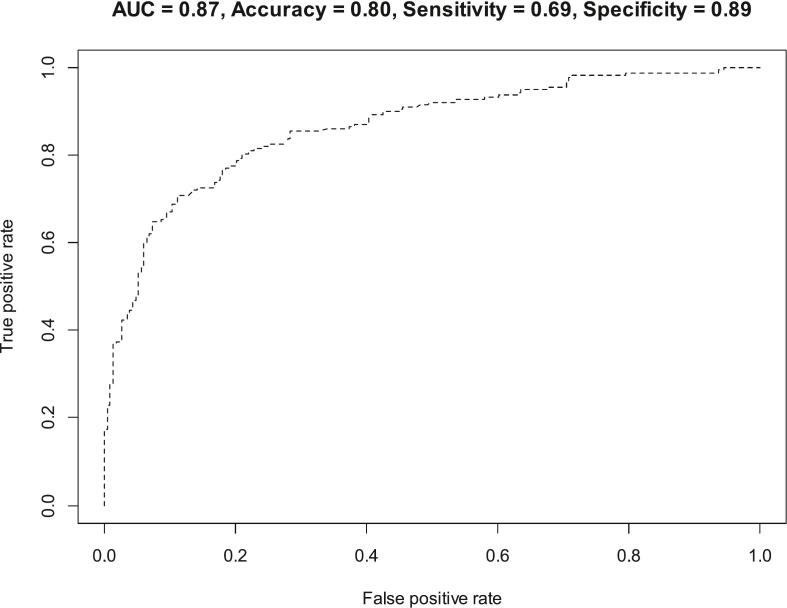
Receiver operative characteristic analysis of the regression model.

The regression model with the predictors was significantly (Chi-squared test statistic = 195.85, p-value<0.001) better than the null model without predictors. Also, the model reported acceptable predictive ability (Fig. 2), and it explains 51 % (Nagelkerke pseudo-R-squared = 51 %) of the variation in *P. falciparum* infection, and classified 80 % of the responses accurately (Fig. 2). Adjusting for other variables, individuals at study sites, such as Bosusu (*p* = 0.045, AOR = 0.23, 95 % CI: 0.05, 0.96), Dwenase (*p* < 0.001, AOR = 0.12, 95 % CI: 0.04, 0.30) and Nsutam (p < 0.001, AOR = 0.19, 95 % CI: 0.08, 0.45) were less likely to contract malaria as compared to those who stayed at Osino. Also, those with no formal education (*p* = 0.046) were less likely to have malaria as compared to those who are still in school. Further, those who frequently stayed outside at night (*p* = 0.001, AOR = 4.64, 95 % CI: 1.97, 11.31) or those who sometimes stayed outside at night (p = 0.001, AOR = 3.77, 95 % CI: 1.76, 8.38) were more likely to have malaria as compared to those who frequently stayed indoors. Moreover, those who had medium housing quality (*p* = 0.031, AOR = 2.31, 95 % CI: 1.09, 5.00) were more likely to have malaria than those who lived in desirable housing quality (Table 4).

**Table 4:** Multiple logistic regression model for predicting *P. falciparum* infection.

Predictors of asymptomatic malaria	AOR	95 % CI	p-value
(Intercept)	96.05	3.19–3385.61	0.010*
Study site [Bosusu community]	0.23	0.05–0.96	0.045*
Study site [Dwenase community]	0.12	0.04–0.30	<0.001*
Study site [Nsutam community]	0.19	0.08–0.45	<0.001*
Gender [Male]	0.93	0.53–1.65	0.817
Marital status [Married]	0.47	0.04–5.30	0.544
Marital status [Single]	0.40	0.03–4.88	0.472
Marital status [minor, (< 18 years)]	0.19	0.01–4.56	0.311
Children [1–12 years]	0.63	0.21–1.86	0.400
Adults [18–65 years]	0.47	0.02–8.74	0.614
Older adults [66 + years]	0.18	0.00–5.86	0.343
Duration of stay in the community [10–19 years]	1.24	0.61–2.52	0.556
Duration of stay in the community [20–29 years]	1.07	0.38–2.96	0.901
Duration of stay in the community [30–39 years]	0.59	0.16–2.02	0.414
Duration of stay in the community [40+ years]	1.70	0.53–5.55	0.374
Education [Below formal education age (< 4 years)]	1.00	0.36–2.73	0.999
Education [Completed school]	0.33	0.10–1.03	0.058
Education [No formal education]	0.22	0.05–0.94	0.046*
Occupation [Unemployed]	0.44	0.02–7.20	0.570
Occupation [Farming related job]	0.53	0.02–9.53	0.674
Occupation [Non-farming related job]	0.72	0.04–10.06	0.807
Mining activities residence [Yes]	1.64	0.49–5.71	0.425
Stay outside long night [At times]	3.77	1.76–8.38	0.001*
Stay outside long night [Yes]	4.64	1.97–11.31	0.001*
Housing quality [Fair]	2.31	1.09–5.00	0.031*
Housing quality [Poor]	1.07	0.48–2.31	0.874
Body temperature [<36 °C]	0.01	0.00–0.05	<0.001*
Body temperature [36–36.5 °C]	0.02	0.01–0.05	<0.001*
Body temperature [36.6–36.9 °C]	0.05	0.02–0.13	<0.001*
Body temperature [37–37.5 °C]	0.17	0.06–0.43	<0.001*

AOR = Adjusted odds ratio, * Significant association.

## 4 Discussion

This study found out that the overall prevalence of asymptomatic malaria in the district was 43.4 %. Compared to Kwahu South (prevalence rate of 11.94 %) (Owusu et al., 2016), a contiguous district, the prevalence rate reported in this current study was significantly higher. This could possibly be due to the forested nature of the Fanteakwa South district, since forest areas provide a conducive environment for the survivability of the vectors (Dakorah et al., 2022). Compared to the Bosusu and Dwenase communities, the prevalence rates of asymptomatic malaria were significantly higher in the Osino communities. This could be due to the presence of artisanal mining activities very close to human habitations. Mining activities have left several pockets of stagnant water, which serve as breeding sites for malaria vectors. It was not surprising to observe that the presence of artisanal mining activities doubled the likelihood of asymptomatic malaria at the study sites. The high turbidity and heavily polluted stagnant water are able to support the life cycle of *Anopheles* mosquitoes. This has been established in past studies in Ghana (Coetzee et al., 2006). It was interesting to note that the mosquitoes bred in these areas were resistant to commonly used insecticides (Coetzee et al., 2006; Dery et al., 2015). Although gender was not associated with asymptomatic malaria, it was observed that women who were not pregnant were at higher risk of asymptomatic malaria compared to pregnant women. This observation could be due to the fact that pregnant women are more likely to be indoors compared to those not pregnant (Franklin et al., 2019) and the free ITN supplied to them for every pregnancy to offer protection against mosquito bites (Nuñez et al., 2023). Furthermore, sulfadoxine-pyrimethamine for intermittent preventive treatment of malaria in pregnancy (IPTp-SP) reduces the risk of malaria in pregnant women (Dosoo et al., 2021; Mikomangwa et al., 2020).

Although the risk of asymptomatic malaria increased, albeit insignificant, among the Ewe ethnic group and among Christians and Moslems, these findings identify essential groups that should be targeted for intensive education on malaria control and prevention. Interestingly, minors less than 18 years of age were more than twice at risk of asymptomatic malaria compared to married couples. This is not surprising since children are known to engage in prolonged frequent outdoor activities (Acheampong et al., 2021), have incompetent anti-malaria immunity (O'Flaherty et al., 2017) and attract mosquitoes to themselves due to increased release of mosquito chemoattractants such as carbon dioxide and lactic acid. This is due to the less dense skin in children and improper skin hygiene (Robinson et al., 2018).

Another interesting observation worth mentioning is that, study participants who were still in school were more likely to be infected asymptomatically with malaria parasites compared to those who completed school. Several reasons could be ascribed to this observation. Firstly, individuals who have completed school have a better appreciation of malaria control and prevention protocols and adhere to them compared to school children. Secondly, overcrowding in classrooms and dormitories could increase the chemoattractant for the malaria vector, which allows mosquitoes to hide underneath classroom and dormitory furniture. Through personal observations, none of the classrooms in the district has fitted mosquito screens together with prominent uncovered eaves. Thirdly, it was also personally observed that there were several stagnant water very close to the schools located at the study sites. The stagnant water pools were left by mining activities in both the rainy and dry seasons.

Despite government efforts to distribute long-lasting insecticide nets (LLIN), free of charge (Nuñez et al., 2023), it was interesting to realise that more than half (54.6 %) of the study participants did not have LLIN. The mosquito net coverage reported in this study (45.4 %) was less than the national average (57.4 %) recently published by Aheto et al. (2023). Among those who had the nets, 63.1 % shared the nets with more than two people. Considering the size of the nets, the integrity of the nets was likely to be distorted when shared and, for that matter, may not protect as desired. Those who shared the nets were more likely to have asymptomatic malaria, with 48.3 % of them having asymptomatic malaria compared to 30.4 % of those who used it alone having malaria.

Another factor associated with asymptomatic malaria was the quality of the housing. Interestingly, those with a medium quality housing were at increased risk of asymptomatic malaria compared to those with low quality houses. The houses with medium quality had three or four of these features; built with mud, with clay flooring, grass-thatched, no ceiling, windows not fitted with nets, and absence of ceiling fans. Dwellers in houses built with clay flooring obviously fall within a low socioeconomic status, an indicator strongly associated with both clinical (Awuah et al., 2018; Kawaguchi et al., 2022) and asymptomatic malaria (Bempah et al., 2020; Mensah et al., 2021) in Ghana. A previous study in Tanzania reported that *An. funestus* and *An. arabiensis* preferred to rest on grassy roofs compared to metal roofs (Msugupakulya et al., 2020). Additionally, buildings without ceilings and windows without fitted nets are associated with increased mosquito invasion in rooms (Animut et al., 2013; Bigira et al., 2015; Lindsay et al., 2003; Temu et al., 2012). The invaded mosquitoes can rest on surfaces and fly around in the room without operational electric ceiling fans.

It was interesting to observe that participants with no formal education were less likely to have asymptomatic malaria as contrasted in a recent study done in the Eastern region of Ghana (Lopez and Brown, 2023). Even though educational level did not associate with malaria transmission in a previous study (Agu and Nwojiji, 2005), persons with higher education have been found to have adequate knowledge on mosquitoes behaviours. This helps them to prevent mosquito bites by accepting the use of mosquito nets and other mosquito control measures (Kudom and Mensah, 2010). The reason only why enrolees without formal education were less likely to have asymptomatic malaria was because they were not schooling. Therefore, they were at similar less likelihood as those that have completed school. Another significant observation made in this study is the association of higher temperatures with asymptomatic malaria. Although this observation may not be new, it conforms to the earlier finding that low grade inflammation is associated with asymptomatic malaria (Mooney et al., 2022). It must be noted that, body temperature elevation alone is not diagnostic of malaria of any kind. Therefore, testing for malaria in cases of hyperthermia is advisable.

## 5 Conclusion

Compared to adjoining districts, the prevalence rate of asymptomatic malaria in the Fanteakwa South district was high (43.4 %). Using molecular assays such as polymerase chain reactions (PCR) and loop-mediated isothermal amplification (LAMP) techniques would have yielded higher rates, because of their comparative sensitivity. Adjusting for other variables, individuals living in Bosusu, Dwenase, and Nsutam were less likely to contract malaria compared to those living in Osino. In addition, the presence of mining activities increased the odds of asymptomatic malaria. Furthermore, study participants who had completed any level of formal education were less likely to have asymptomatic malaria compared to those who were still in school. Those who had regular prolonged nocturnal outdoor activity or stayed outside at night were more likely to have asymptomatic malaria compared to those who frequently stayed indoors at night. In addition, those who had a medium housing quality were more likely to have malaria than those who had desirable housing quality. Taking into account the preceding, malaria control strategies should be targeted at Osino residents, residents of mining communities, and individuals still in school. Furthermore, residents of the communities should be educated to remain indoors at night and improve the quality of their dwelling places.

## CRediT authorship contribution statement

**Enoch Aninagyei:** Conceptualization, Methodology, Project administration, Supervision, Validation, Writing – original draft. **David Adedia:** Data curation, Formal analysis, Validation, Visualization, Writing – review & editing. **Gifty Larbi:** Formal analysis, Investigation, Methodology, Writing – review & editing. **Stella Omane Acheampong:** Data curation, Formal analysis, Funding acquisition, Visualization, Writing – review & editing. **Margaret Nyarko:** Conceptualization, Project administration, Supervision, Writing – review & editing. **George Abeiku Abbew:** Investigation, Methodology, Visualization, Writing – review & editing. **Isaac Tuwarlba:** Investigation, Methodology, Writing – review & editing. **Desmond Omane Acheampong:** Conceptualization, Funding acquisition, Investigation, Methodology, Supervision, Writing – review & editing.

## Declaration of competing interest

The authors declare no conflict of interest.
